# Outdoor Air Pollution and Indoor Window Condensation Associated with Childhood Symptoms of Allergic Rhinitis to Pollen

**DOI:** 10.3390/ijerph19138071

**Published:** 2022-06-30

**Authors:** Yingjie Liu, Chan Lu, Yuguo Li, Dan Norbäck, Qihong Deng

**Affiliations:** 1 School of Energy Science and Engineering, Central South University, Changsha 410083, China; yingjieliu@csu.edu.cn; 2 XiangYa School of Public Health, Central South University, Changsha 410078, China; chanlu@csu.edu.cn; 3 Department of Mechanical Engineering, The University of Hong Kong, Hong Kong 999077, China; liyg@hku.hk; 4 Department of Medical Sciences, Uppsala University, 752 36 Uppsala, Sweden; dan.norback@medsci.uu.se; 5 School of Public Health, Zhengzhou University, Zhengzhou 450001, China

**Keywords:** seasonal allergic rhinitis, pollen allergy, air pollution, window condensation, indoor ventilation

## Abstract

Pollen is the main factor causing asthma and allergic rhinitis (AR). However, the key indoor and outdoor factors associated with childhood symptoms of allergic rhinitis (SAR) to pollen are unclear. We investigate the association of exposure to outdoor air pollution and indoor environmental factors with childhood SAR to pollen and consider SAR to pollen in different seasons. A cross-sectional study of 2598 preschool children aged 3–6 was conducted in Changsha, China (2011–2012). The prevalence of SAR to pollen in children and information on indoor environmental factors were obtained by questionnaire. Children’s exposure to outdoor air pollutants (PM_10_, SO_2,_ and NO_2_) was estimated from the monitored concentrations. The association of exposure to indoor environmental factors and outdoor air pollution with childhood SAR to pollen was estimated by multiple logistic regression models using odds ratio (OR) and a 95% confidence interval (CI), and the relationship between outdoor air pollutants and childhood SAR to pollen was investigated using restricted cubic splines. We found that early-life and current exposure to outdoor air pollution were significantly associated with childhood SAR to pollen in autumn, including exposure to SO_2_ one year before conception (OR = 1.60, 95% CI = 1.08–2.37) and during entire pregnancy (OR = 1.49, 95% CI = 1.01–2.20) periods, exposure to PM_10_ during the current period (OR = 1.78, 95% CI = 1.07–2.96), and exposure to NO_2_ during the early-life (one year before conception and entire pregnancy) and current periods with ORs (95% CI) of 1.72 (1.10–2.71), 1.82 (1.17–2.83), and 1.94 (1.11–3.40), respectively. Further, we found significant associations of both prenatal and postnatal exposure to window condensation with childhood SAR to pollen, with ORs (95% CI) = 1.37 (1.05–1.77) and 1.38 (1.02–1.88), respectively. We encourage SAR to pollen sufferers to stay indoors due to outdoor air pollution and higher pollen concentration outdoors, but indoor ventilation should be maintained.

## 1. Introduction

As one of the most common chronic diseases [1], allergic rhinitis (AR) affects more than 40% of the world’s population [2], and its prevalence is rising rapidly in low- and middle-income countries [3]. AR in children receives considerable attention due to its high economic burden on families and substantial impairment of children’s quality of life and their learning and performance at school [4]. Plant pollen is considered a major cause of asthma and AR [5,6]. The rapid development of information and communication technologies and the urban economy have contributed to an accelerating urbanization process in the last few decades [7]. Urban green spaces are shrinking due to accelerated urbanization [8]. However, symptoms of allergic rhinitis (SAR) to pollen and respiratory allergy symptoms are still increasing, and the prevalence of pollen-induced respiratory allergies in urban areas is higher than that in rural areas [9], which is mainly due to the role of outdoor air pollution [10,11] and indoor environmental factors.

Recent studies have suggested that exposure to air pollution may cause the development and/or symptom exacerbation of AR in children. Both epidemiological studies and toxicological experiments have consistently found that exposure to air pollution can contribute to AR [12,13,14,15,16]. An increasing number of studies have shown that air pollution modifies the effect of pollen on allergic respiratory symptoms and diseases [17,18]. Experimental studies have provided evidence that air pollution increases pollen allergen release and modifies the allergenic potential of pollen grains [11,19]. Many epidemiological studies have shown that the interaction of pollen and air pollutants increases the risk of allergic reactions [20,21,22,23]. However, there are very few studies that investigate the effect of exposure to early-life and current air pollution on childhood SAR to pollen, especially in different seasons.

Indoor air pollutant levels have increased due to rapid urbanization [24], and we spend more than 90 percent of our time indoors [25]. Many epidemiological studies have shown that indoor environmental factors are associated with AR in children and young adults, including indoor visible mold/damp stains [25,26,27,28,29] and window condensation [26,28]. There are studies that have found that moldy odor [30] and window condensation [31] can increase the risk of hay fever. However, the key indoor environmental risk factors for the development of SAR to pollen in children are unclear.

With the rapid urbanization and development of the economy during the past few decades, China has witnessed serious indoor and outdoor air pollution [32,33,34]. China’s air quality has improved recently [35], but the impact of air pollution on health is irreversible. We aimed to explore whether exposure to indoor environmental factors (in the prenatal and postnatal periods) and outdoor air pollution (in the early-life and current periods) has increased the risk of SAR to pollen in children, especially the risk of SAR to pollen in different seasons.

## 2. Materials and Methods

### 2.1. Study Protocol and Questionnaire

A cross-sectional study was carried out in Changsha during the period from September 2011 to January 2012 as part of the nationwide “China-Children-Homes-Health (CCHH)” study [3]. The cross-sectional study protocol was approved by the Ethics Committee of Central South University and has been described in detail elsewhere [36]. In short, we used a standard questionnaire from the International Study of Asthma and Allergies in Childhood (ISAAC) [37] to obtain information about allergic diseases in children, and a Swedish questionnaire about dampness in buildings and health (DBH) [38] was used to survey the lifestyle and indoor environment of the children and their families. A total of 4988 questionnaires were distributed to children from 36 randomly selected kindergartens. We asked the parents to fill out the questionnaire and return them to the kindergarten within a week. A total of 3897 completed questionnaires were received, of which 2598 from children 3–6 years old were finally used in this work.

### 2.2. Exposure Windows

We divided the timing windows for indoor environmental factor exposure into prenatal and postnatal periods. The prenatal period was defined as the period from the first month to the last month of pregnancy. We divided timing windows for outdoor air pollution exposure into the early-life (one year before conception and prenatal) and current periods. The prenatal period can be divided into three gestational periods: months 1–3 (the first trimester), months 4–6 (the second trimester), and months 7–last (the third trimester), respectively. The current period was defined as the past 12 months or recent year(s).

### 2.3. Exposure to Indoor Environmental Factors 

Both prenatal and postnatal exposure to indoor environmental factors included environmental tobacco smoke (ETS), new furniture, house redecoration, visible mold/damp stains at home, and window condensation. Detailed information on these factors is given in Table S1.

### 2.4. Exposure to Outdoor Air Pollution

Three types of air pollutants, sulfur dioxide (SO_2_), nitrogen dioxide (NO_2_), and particulate matter with a diameter ≤ 10 μm (PM_10_), were selected as indicators of industry-related air pollution (IRAP), traffic-related air pollution (TRAP), and mixed air pollution, respectively [39]. The daily (24 h) average concentration of air pollutants was obtained from seven monitoring stations, and using these data, each child’s daily exposure was estimated using the inverse distance weighted (IDW) method [39]. The prenatal exposure of children was calculated as the average monthly concentrations of PM_10_, SO_2_, and NO_2_ during the early-life (one year before conception and prenatal) and current periods.

### 2.5. Health Outcome

The health outcome in this study was the symptoms of allergic rhinitis (SAR) to pollen, which was based on the answer to the question: “During past 12 months, whether your child had the rhinitis-like and eyes symptoms including sneezing, run-ning nose, stuffy nose, eyes’ pain and tears after contacting with pollen or plants? If so, which month?” It was a modification of the original ISACC question for investigating SAR to pollen, especially in different seasons. The original question from ISAAC reads as follows: “In the past 12 months, has your child had a problem with sneezing, or a runny, or a blocked nose when he/she DID NOT have a cold or the flu?”. SAR to pollen in different seasons was divided into spring (March to May), summer (June to August), autumn (September to November), and winter (December to February).

### 2.6. Confounding Covariates

Potential confounding variables were obtained from the questionnaire filled in by parents, which included personal factors (child’s sex, age, birth season, breast-feeding, antibiotics use, and parental atopy) and indoor environmental factors (ETS at home, incense used, visible mold/damp stains, window condensation in winter, air humidifiers, cockroaches noted, and household pets) (Table S1) [25,26].

### 2.7. Statistical Analysis

The associations between childhood SAR to pollen and indoor environmental factors as well as outdoor air pollution were evaluated using multiple logistic regression models in terms of the odds ratio (OR) and 95% confidence interval (95% CI). The association between childhood SAR to pollen and exposure to indoor environmental factors was estimated with the classification model, and the OR was estimated in comparison with no exposure to indoor environmental factors. The relationship between childhood SAR to pollen and exposure to outdoor air pollutants was examined with continuous models. The OR (95% CI) in the continuous model was estimated with the interquartile range (IQR) increase in each pollutant exposure. We used restricted cubic spline regressions with four knots based on multiple logistic regression models for outdoor air pollution to model concentration–response curves. The association was considered statistically significant if the value of *p* was less than 0.05 (*p* < 0.05). All statistical analyses were performed using SPSS software (version 16.0, SPSS Inc., Chicago, IL, USA) and R software (version 4.0.5; RStudio, Boston, MA, USA).

## 3. Results

Of the 2598 children, 318 (12.2%) had symptoms of allergic rhinitis (SAR) to pollen in the previous 12 months. Table 1 shows the prevalence of SAR to pollen stratified by covariates. We found that children with parental atopy (21.1%) had significantly higher prevalence than those without parental atopy (11.2%) (*p* < 0.05). The prevalence of SAR to pollen was significantly higher among children who lived in a dwelling with mold/damp stains, window condensation, and incense used than among those living in a dwelling without mold/damp stains, window condensation, and incense used (*p* < 0.05). We further observed a higher prevalence of SAR to pollen in children with antibiotics used (12.9%) compared to children without antibiotics used (9.7%). Some families kept household pets that were found to have a significant effect on SAR to pollen. However, no difference in prevalence was observed among children stratified by the other covariates, including sex, age, birth season, breast-feeding, house size, ETS at home, house redecoration, and window condensation in winter.

The associations between children with SAR to pollen and exposure to outdoor air pollution are shown in Table 2. Exposure to SO_2_ in the first trimester of pregnancy was significantly associated with SAR to pollen in children, with an adjusted OR (95% CI) = 1.18 (1.02–1.37).

Table 3 provides the association between exposure to outdoor air pollution during the early-life and current periods and children with SAR to pollen in different seasons. We found a significant association between current exposure to PM_10_ and children with autumn SAR to pollen, with an OR (95% CI) = 1.78 (1.07–2.96). Early-life exposure to SO_2_ was significantly associated with autumn SAR to pollen in children, with an OR (95% CI) = 1.60 (1.08–2.37) during one year before conception, an OR (95% CI) = 1.42 (1.02–1.96) during the first trimester, an OR (95% CI) = 1.35 (1.03–1.77) during the second trimester, and an adjusted OR (95% CI) = 1.49 (1.01–2.20) during the entire pregnancy period. We further found that both early-life and current exposure to NO_2_ were significantly associated with childhood autumn SAR to pollen, with ORs (95% CI) = 1.72 (1.10–2.71), 1.60 (1.02–2.49), 1.90 (1.22–2.96), 1.82 (1.17–2.83), and 1.94 (1.11–3.40) during one year before conception, the second and third trimester, the entire pregnancy, and current period, respectively. However, the third trimester and entire pregnancy exposure to PM_10_ were significantly associated with winter SAR to pollen in children, with ORs (95% CI) = 0.45 (0.25–0.82) and 0.48 (0.26–0.90), respectively. Associations between pregnancy exposure to outdoor air pollution and childhood SAR to pollen in the multi-pollutant model and multi-pollutant/window model were the same as that in the single-adjusted model (Tables S3 and S4).

**Figure 1:**
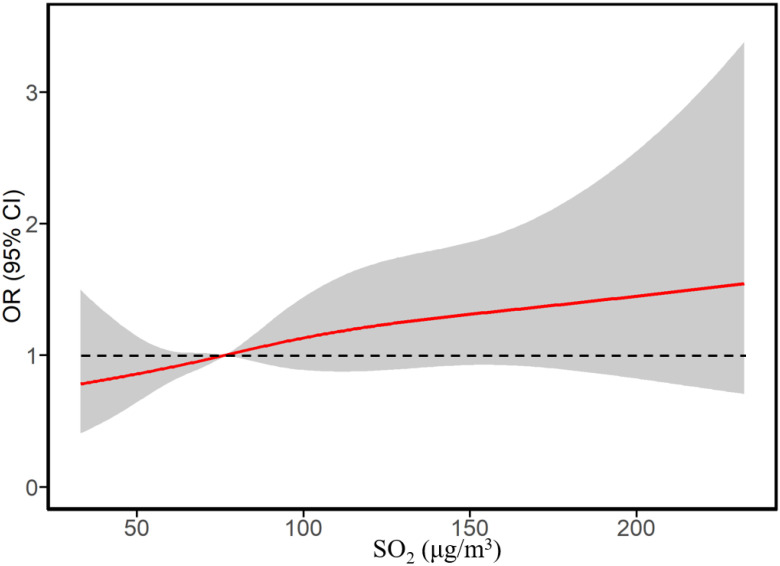
Concentration–response curves between exposure to SO_2_ in the 1st trimester and risks of childhood annual SAR to pollen. OR (95% CI) was adjusted for all the covariates in Table 1.

**Figure 2:**
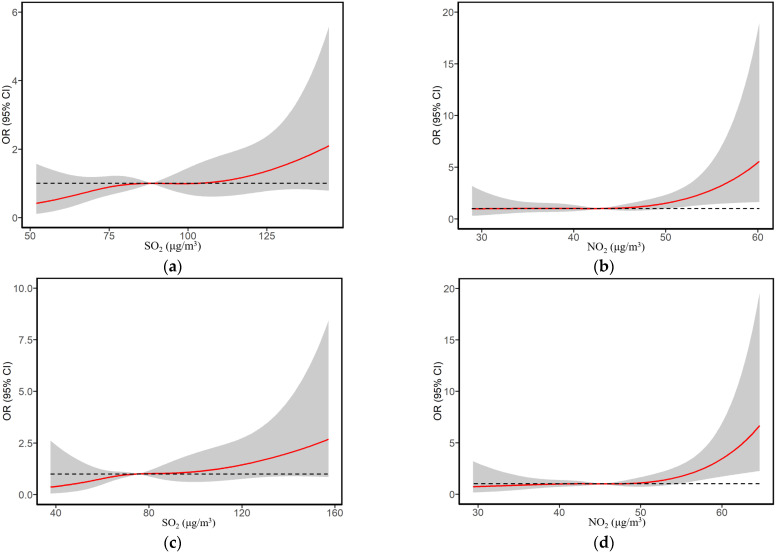

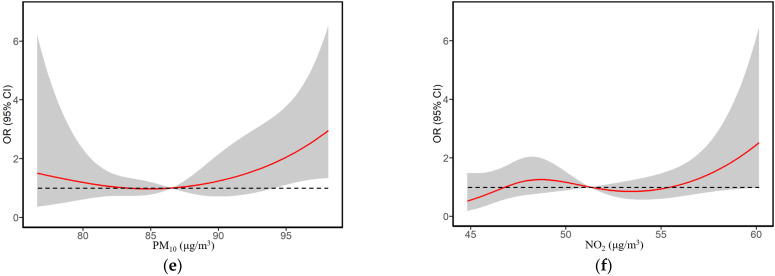
Concentration–response curves between exposure to PM_10_, SO_2_, and NO_2_ during time windows and risks of childhood autumn SAR to pollen. OR (95% CI) was adjusted for all the covariates in Table 1. (**a**) One year before conception SO_2_. (**b**) One year before conception NO_2_. (**c**) Entire pregnancy SO_2_. (**d**) Entire pregnancy NO_2_. (**e**) Current PM_10_. (**f**) Current NO_2_.

We assessed the continuous association between exposure to SO_2_ in the first trimester and the risk of childhood SAR to pollen using a restricted cubic spline curve (Figure 1). A relatively linear relationship was observed between the risk of childhood SAR to pollen and first trimester SO_2_ exposure. Figure 2 illustrates concentration–response curves between autumn SAR to pollen in children and SO_2_ exposure during the early-life period, PM_10_ exposure during the current period, and NO_2_ exposure during both the early-life and current periods. All figures showed linear increases in risks associated with in PM_10_, SO_2,_ and NO_2_ exposure during the early-life and current periods.

We found that prenatal and postnatal exposure to window condensation were significantly associated with childhood SAR to pollen, with adjusted ORs (95% CI) = 1.37 (1.05–1.77) and 1.38 (1.02–1.88) (Table 4), respectively. We further found a significant association between exposure to visible mold/damp stains during prenatal period and SAR to pollen in children, with a crude OR (95% CI) = 1.41 (1.01–1.96).

## 4. Discussion

This is the first cross-sectional study to examine the risk of prenatal exposure to outdoor air pollution and indoor environmental factors on the symptoms of allergic rhinitis (SAR) to pollen in children. We found that early-life and current exposure to outdoor air pollution (PM_10_, SO_2_, and NO_2_) were significantly associated with childhood SAR to pollen in autumn. We further found a significant association of exposure to window condensation during prenatal and postnatal periods with SAR to pollen in children. Studies have reported that there is less condensation on the windows of rooms with a higher air change rate [40,41]. Therefore, we suggested that ventilation indoors may help reduce the risk of childhood SAR to pollen.

We found that exposure to SO_2_ during the third trimester of pregnancy was significantly associated with SAR to pollen in children, and in particular, we found a significant relationship between early-life and current exposure to outdoor air pollution (PM_10_, SO_2_ and NO_2_) and childhood SAR to pollen in autumn. There is increasing evidence of a causal relationship between outdoor air pollution and the risk of pollen-related allergic diseases, especially in children. A study found that exposure to both PM_2.5_ and NO_2_ in highway tunnels may significantly enhance asthmatic reactions to inhaled pollen allergens [23]. A prospective birth cohort study reported that exposure to PM_2.5_ significantly increased the risk of hay fever (OR, 1.59; 95% CI, 1.11–2.27) and allergic sensitization to pollen (OR, 1.40; 95% CI, 1.20–1.64) in children [21]. A European birth cohort study observed that sensitization to inhaled pollen allergens may be related to PM_2.5_ and NO_2_ [20]. Many studies have shown that the interaction between air pollutants and pollens might promote the release of pollen allergens, modify the potential of allergens, and enhance the expression of some allergens in pollen grains [11]. Moreover, studies have found that autumn is one of the two peaks of AR [42,43,44]. Symptomatic AR in autumn may be caused by weed pollen allergens [45] and the increasing prevalence of weed pollen allergy in children [46]. One study observed a significant positive association between air pollutants (SO_2_), pollens, and the number of outpatients for AR during autumn (43.00967 ± 0.11284, *p* < 0.001) [47]. The different association of exposure to PM_10_ during the third trimester and the entire pregnancy with winter SAR to pollen in children may be because of the low case numbers of childhood SAR to pollen in winter. 

We observed a significant association between exposure to window condensation during the prenatal and postnatal periods and childhood SAR to pollen. Although we have not found identical studies to ours, a large number of studies support our findings. Studies have observed that window condensation may increase the risk of childhood SAR [26,28]. One study indicated that window condensation may increase the risk of airway infection during the past 12 months (OR = 1.66; 95% CI 1.17–2.37) and allergy to pollen (OR = 1.54; 95% CI 1.15–2.06) [31]. Window condensation is a proxy for less ventilation indoors [40,41]. Thus, our findings showed that indoor ventilation might be associated with SAR to pollen in children.

When staying outdoors, high levels of pollen and outdoor air pollution exposure might increase the risk of SAR to pollen. However, staying indoors without ventilation may also be a risk factor. The possible reason is that high pollen levels may increase the risk of SAR to pollen, while appropriate ventilation indoors might enrich indoor air microbiomes that reduce pollen allergy response. It is known to all that the allergic symptoms due to pollen become more frequent and serious with an increase in pollen counts that exceed a certain threshold [48,49,50]. Studies have shown that ventilation is the key factor in controlling indoor microbiomes [51] and that outdoor air is one of the main contributors to indoor airborne microbiomes [52]. The biodiversity hypothesis states that the abundance of human microbiota promotes immune balance and protects from allergic diseases [53,54].

There are some limitations that should be acknowledged in this study. On the one hand, SAR to pollen was not assessed by skin prick testing or measuring serum IgE. Therefore, some symptomatic responses to AR due to pollen in children may have been missed. On the other hand, we did not distinguish between specific types of pollen in our questionnaire, and thus we could not analyze which factors play key roles in SAR due to a specific type of pollen. We will consider the above limitations in a future study on SAR to pollen in children.

## 5. Conclusions

Our study found that exposure to outdoor air pollution during the early-life and current periods was significantly associated with the prevalence of childhood SAR to pollen in autumn. Meanwhile, prenatal and postnatal exposure to window condensation significantly increased the risk of SAR to pollen in children. Due to global climate change and air pollution, the harm of pollen sensitization has been aggravated. Meanwhile, outdoor air pollution in developing countries is still serious. We provide a measure to reduce the risk of SAR to pollen during pollen season when outdoor air pollution is high, which is to stay indoors and maintain good ventilation.

## Figures and Tables

**Table 1:** Demographic information and prevalence of SAR to pollen among children (*n* = 2598).

	*n*	Case	Prevalence (%)	*p*-Value
Total	2598	318	12.2	—
**Sex**				
Boys	1399	181	12.9	0.246
Girls	1199	137	11.4	
**Age (years)**				
3	665	82	12.3	0.898
4	952	114	12.0	
5	815	104	12.8	
6	166	18	10.8	
**Birth season**				
Warm (May–September)	1152	149	12.9	0.327
Cold (October–April)	1446	169	11.7	
**Breast-feeding**				
No	222	26	11.7	0.807
Yes	2376	292	12.3	
**Antibiotics used**				
No	432	42	9.7	0.059
Yes	2115	273	12.9	
**Parental atopy**				
No	2332	262	11.2	**<0.001**
Yes	266	56	21.1	
**Environmental tobacco smoke (ETS) at home**				
No	864	96	11.1	0.217
Yes	1734	222	12.8	
**Incense used**				
No	1845	204	11.1	**0.005**
Yes	705	107	15.2	
**Visible mold/damp stains**				
No	1985	227	11.4	**0.027**
Yes	606	90	14.9	
**Window condensation in winter**				
No	1175	116	9.9	**<0.001**
Yes	1369	201	14.7	
**Air humidifier**				
No	2095	263	12.6	0.316
Yes	277	41	14.8	
**Cockroaches**				
No	827	97	11.7	0.487
Yes	1653	210	12.7	
**Household pets**				
No	2300	292	12.7	0.061
Yes	294	26	8.8	

Sum of number is not 2598 due to the missing data. The *p*-values < 0.05 are in bold.

**Table 2:** Odds ratio (95% CI) of childhood annual SAR to pollen for exposure to outdoor air pollution during 1 year before conception, prenatal, and current periods (*n* = 2598).

	Crude OR	Adjusted OR #
**1 year before conception**		
PM_10_	0.97 (0.82–1.16)	1.01 (0.72–1.42)
SO_2_	1.02 (0.85–1.22)	1.11 (0.91–1.36)
NO_2_	1.02 (0.84–1.23)	1.10 (0.88–1.39)
**Prenatal**		
**1st trimester**		
PM_10_	1.08 (0.93–1.24)	1.09 (0.89–1.33)
SO_2_	1.11 (0.98–1.26)	1.18 (1.02–1.37) *
NO_2_	1.07 (0.90–1.27)	1.09 (0.87–1.37)
**2nd trimester**		
PM_10_	0.92 (0.80–1.06)	0.91 (0.77–1.07)
SO_2_	1.05 (0.94–1.18)	1.08 (0.95–1.24)
NO_2_	0.98 (0.84–1.16)	0.97 (0.79–1.20)
**3rd trimester**		
PM_10_	0.92 (0.81–1.05)	0.89 (0.74–1.06)
SO_2_	0.93 (0.81–1.07)	0.99 (0.83–1.17)
NO_2_	0.94 (0.79–1.11)	0.98 (0.80–1.20)
**Entire pregnancy**		
PM_10_	0.94 (0.83–1.08)	0.89 (0.72–1.09)
SO_2_	1.05 (0.89–1.23)	1.16 (0.95–1.41)
NO_2_	0.99 (0.83–1.19)	1.02 (0.82–1.28)
**Current**		
PM_10_	0.94 (0.83–1.08)	1.16 (0.90–1.51)
SO_2_	1.05 (0.89–1.23)	1.24 (0.99–1.56)
NO_2_	0.99 (0.83–1.19)	1.07 (0.81–1.41)

OR (95% CI) was estimated for an IQR increase in PM_10_, SO_2_, and NO_2_. # Models were adjusted for all the covariates in Table 1. * *p* ≤ 0.05.

**Table 3:** Odds ratio (95% CI) of childhood seasonal SAR to pollen for exposure to outdoor air pollution during 1 year before conception, prenatal, and current periods (*n* = 2598).

	SAR to Pollen			
	Spring	Summer	Autumn	Winter
**1 year before conception**				
PM_10_	1.32 (0.86–2.03)	0.88 (0.43–1.81)	1.06 (0.55–2.06)	0.67 (0.27–1.65)
SO_2_	0.98 (0.76–1.26)	0.93 (0.61–1.43)	1.60 (1.08–2.37) **	1.16 (0.65–2.07)
NO_2_	0.91 (0.68–1.21)	1.10 (0.68–1.77)	1.72 (1.10, 2.71) **	1.28 (0.66–2.45)
**Prenatal**				
**1st trimester**				
PM_10_	1.02 (0.78–1.33)	1.33 (0.87–2.05)	1.05 (0.70–1.58)	0.98 (0.56–1.72)
SO_2_	1.06 (0.85–1.33)	1.22 (0.86–1.73)	1.42 (1.02–1.96) **	1.57 (0.97–2.54)
NO_2_	0.85 (0.62–1.17)	1.01 (0.60–1.70)	1.56 (0.96, 2.54)	1.22 (0.58–2.55)
**2nd trimester**				
PM_10_	0.93 (0.73–1.19)	0.91 (0.61–1.37)	0.98 (0.67–1.44)	0.82 (0.48–1.42)
SO_2_	1.13 (0.93–1.38)	1.05 (0.77–1.45)	1.35 (1.03–1.77) **	1.30 (0.87–1.92)
NO_2_	0.86 (0.64–1.15)	0.81 (0.49–1.34)	1.60 (1.02–2.49) **	1.20 (0.62–2.33)
**3rd trimester**				
PM_10_	0.89 (0.70–1.13)	0.93 (0.63–1.39)	0.83 (0.57–1.22)	0.45 (0.25–0.82) **
SO_2_	1.07 (0.83–1.38)	0.78 (0.49–1.23)	1.17 (0.81–1.69)	0.96 (0.58–1.60)
NO_2_	0.90 (0.67–1.21)	0.74 (0.45–1.21)	1.90 (1.22–2.96) **	0.88 (0.46–1.69)
**Entire pregnancy**				
PM_10_	0.87 (0.66–1.15)	1.04 (0.65–1.65)	0.91 (0.59–1.40)	0.48 (0.26–0.90) **
SO_2_	1.13 (0.87–1.47)	1.06 (0.69–1.62)	1.49 (1.01–2.20) **	1.33 (0.76–2.33)
NO_2_	0.86 (0.64–1.14)	0.87 (0.54–1.41)	1.82 (1.17–2.83) **	1.02 (0.53–1.95)
**Current**				
PM_10_	0.99 (0.71–1.37)	1.14 (0.66–1.96)	1.78 (1.07–2.96) **	1.11 (0.53–2.33)
SO_2_	1.13 (0.86–1.51)	1.37 (0.85–2.22)	1.49 (0.94–2.37)	0.96 (0.51–1.82)
NO_2_	0.86 (0.60–1.23)	1.06 (0.59–1.92)	1.94 (1.11–3.40) **	1.21 (0.54–2.72)

OR (95% CI) was estimated for an IQR increase in PM_10_, SO_2_, and NO_2_. Models were adjusted for all the covariates in Table 1. ** *p* ≤ 0.01.

**Table 4:** Odds ratio (95% CI) of childhood annual SAR to pollen for exposure to indoor environmental factors during prenatal and postnatal periods (*n* = 2598).

	*n*	Case	(%)	Crude OR	Adjusted OR #
**Prenatal**					
ETS at home	1252	157	(12.5)	1.04 (0.83–1.32)	1.10 (0.85–1.42)
New furniture	338	46	(13.6)	1.17 (0.83–1.65)	1.07 (0.73–1.56)
House redecoration	139	18	(12.9)	1.07 (0.64–1.80)	1.01 (0.58–1.77)
Visible mold/damp stains	435	63	(14.5)	1.26 (0.93–1.69)	1.19 (0.85–1.65)
Window condensation	1222	181	(14.8)	1.54 (1.21–1.96) ***	1.37 (1.05–1.77) *
**Postnatal**					
ETS at home	1734	222	(12.8)	1.17 (0.91–1.52)	1.32 (0.99–1.74)
New furniture	980	136	(13.9)	1.26 (0.99–1.60)	1.22 (0.94–1.59)
House redecoration	499	67	(13.4)	1.14 (0.85–1.52)	1.13 (0.82–1.55)
Visible mold/damp stains	308	49	(15.9)	1.41 (1.01–1.96) *	1.23 (0.85–1.79)
Window condensation	869	139	(16.0)	1.52 (1.15–2.02) **	1.38 (1.02–1.88) *

# Models were adjusted for all the covariates in Table 1. * *p* ≤ 0.05. ** *p* ≤ 0.01. *** *p* ≤ 0.001.

## Data Availability

The data presented in this study are available on request from the corresponding author.

## Supplementary Materials

The following supporting information can be downloaded at: https://www.mdpi.com/article/10.3390/ijerph19138071/s1, Table S1. Descriptions and questions about confounding variables related to childhood symptoms of allergic rhinitis (SAR) to pollen in this study. Table S2. Descriptive statistics for air pollution levels attributed to the children during 1 year before conception, prenatal and current periods (n = 2598). Table S3. Odds ratio (95%CI) of childhood SAR to pollen for exposure to outdoor air pollution during different trimesters of pregnancy (n = 2598). Table S4. Odds ratio (95%CI) of childhood autumn SAR to pollen for exposure to outdoor air pollution during different trimesters of pregnancy (n = 2598). Table S5. Pearson correlations between outdoor air pollutants during different time windows (*n* = 2598).
