# Supplementary material for: Outdoor Air Pollution and Indoor Window Condensation Associated with Childhood Symptoms of Allergic Rhinitis to Pollen
Source: Int J Environ Res Public Health. 2022 Jun 30;19(13):8071. doi: 10.3390/ijerph19138071 (PMC9266097; doi:10.3390/ijerph19138071)
Supplement: Supplementary file 1 [file ijerph-19-08071-s001.zip › ijerph-1757231-supplementary.pdf]

# Supplemental Materials

**Table S1.** Descriptions and questions about confounding variables related to childhood symptoms of allergic rhinitis (SAR) to pollen in this study.

| **Confounding Covariates** | **Questions** |
|---|---|
| Personal factors | |
| Sex | Is your child girl or boy? |
| Age (years) | How old is your child? |
| Birth season | In what season was your child born? (Spring/Summer/Autumn/Winter) |
| Breast-feeding | How long was your child breastfed or fed breast milk? (Never / <1 month / 1–3 months / 3–6 months />6 months) |
| Antibiotics used | Has your child been treated with antibiotics? |
| Parental atopy | Whether the mother or father has a history of allergic rhinitis and/or asthma? |
| Indoor environmental factors | |
| Environmental tobacco smoke (ETS) at home | Did anyone who was living in the same house with you smoke? |
| Indoor new furniture | Did you install new furniture in your residence recently? |
| House redecoration | Have you decorated your home recently? |
| Visible mold/damp stains at home | Have you noticed visible mold or damp stains on the floor, walls, or ceilings of your residence? |
| Window condensation in winter | In winter, have you noticed condensation or water vapor at the bottom of the inside of the window in the room where your child slept? |
| Incense used | Do you use incense in your residence? |
| Air humidifier | Do you use air humidifier in your residence? |
| Cockroaches noted | Have you noted cockroaches in your residence? |
| Household pets | Do you keep pets in your residence? |

**Table S2.** Descriptive statistics for air pollution levels attributed to the children during 1 year before conception, prenatal and current periods ($n$ = 2598).

| | Mean (SD) | 25th | 50th | 75th | IQR |
|---|---|---|---|---|---|
| **1 year before conception** | | | | | |
| $PM_{10}$ | 119 (13) | 109 | 116 | 128 | 19 |
| $SO_2$ | 91 (25) | 70 | 88 | 108 | 38 |
| $NO_2$ | 43 (7) | 38 | 43 | 50 | 12 |
| **Prenatal** | | | | | |
| **1st trimester** | | | | | |
| $PM_{10}$ | 113 (16) | 102 | 111 | 121 | 19 |
| $SO_2$ | 86 (38) | 60 | 77 | 103 | 43 |
| $NO_2$ | 45 (11) | 37 | 44 | 53 | 16 |
| **2nd trimester** | | | | | |
| $PM_{10}$ | 110 (15) | 101 | 109 | 118 | 17 |
| $SO_2$ | 82 (36) | 60 | 73 | 95 | 35 |
| $NO_2$ | 46 (11) | 39 | 46 | 54 | 15 |
| **3rd trimester** | | | | | |
| $PM_{10}$ | 108 (18) | 97 | 107 | 116 | 19 |
| $SO_2$ | 79 (38) | 52 | 71 | 94 | 42 |
| $NO_2$ | 46 (10) | 38 | 45 | 53 | 15 |
| **Entire pregnancy** | | | | | |
| $PM_{10}$ | 110 (11) | 103 | 108 | 115 | 12 |
| $SO_2$ | 82 (26) | 62 | 75 | 98 | 36 |
| $NO_2$ | 46 (8) | 40 | 45 | 52 | 12 |
| **Current** | | | | | |
| $PM_{10}$ | 87 (6) | 81 | 87 | 93 | 12 |
| $SO_2$ | 42 (5) | 39 | 43 | 48 | 9 |
| $NO_2$ | 51 (5) | 46 | 51 | 57 | 11 |

$PM_{10}$ (μg/m$^3$) = particulate matter ≤ 10 μm in aerodynamic diameter; $SO_2$ (μg/m$^3$) = sulfur dioxide; $NO_2$ (μg/m$^3$) = nitrogen dioxide; SD = standard deviation; IQR = interquartile range.

**Table S3.** Odds ratio (95%CI) of childhood SAR to pollen for exposure to outdoor air pollution during different trimesters of pregnancy (n = 2598).

| | **Multi-Pollutant Model †** | | | | **Multi-Pollutant +** |
|---|---|---|---|---|---|
| | **$PM_{10}$ + $SO_2$** | **$PM_{10}$ + $NO_2$** | **$SO_2$ + $NO_2$** | **$PM_{10}$ + $SO_2$ + $NO_2$** | **Window Model ‡** |
| **1st trimester** | | | | | |
| $PM_{10}$ | 1.04 (0.84–1.28) | 1.07 (0.87–1.32) | — | 1.06 (0.86–1.31) | 1.06 ( 0.85–1.31) |
| $SO_2$ | 1.17 (1.00–1.37)* | — | 1.36 (1.06, 1.75)* | 1.36 (1.07–1.74)* | 1.34 ( 1.05–1.72)* |
| $NO_2$ | — | 1.07 (0.85–1.35) | 0.76 (0.51, 1.11) | 0.75 (0.52–1.09) | 0.76 ( 0.51–1.13) |
| **2nd trimester** | | | | | |
| $PM_{10}$ | 0.87 (0.74–1.04) | 0.91 (0.76–1.08) | — | 0.90 (0.75–1.07) | 0.91 ( 0.76–1.09) |
| $SO_2$ | 1.12 (0.97–1.29) | — | 1.22 (1.00–1.48) | 1.23 (1.01–1.50)* | 1.19 ( 0.96–1.48) |
| $NO_2$ | — | 1.02 (0.81–1.28) | 0.78 (0.58–1.07) | 0.82 (0.60–1.12) | 0.85 ( 0.59–1.23) |
| **3rd trimester** | | | | | |
| $PM_{10}$ | 0.88 (0.73–1.06) | 0.87 (0.72–1.06) | — | 0.87 (0.72–1.06) | 0.88 ( 0.72–1.07) |
| $SO_2$ | 1.02 (0.86–1.22) | — | 0.99 (0.76–1.30) | 0.99 (0.76–1.29) | 0.99 ( 0.74–1.31) |
| $NO_2$ | — | 1.04 (0.84–1.30) | 0.99 (0.72–1.35) | 1.06 (0.76–1.47) | 1.11 ( 0.76–1.61) |

OR (95%CI) was estimated for an IQR increase in $PM_{10}$, $SO_2$, and $NO_2$.
Single-adjusted model: Models were adjusted for all covariates in Table 1.
† Multi-pollutant model: Models were further adjusted for the other air pollutants during the same time window based on single-adjusted model.
‡ Multi-pollutant + window model: Models were further adjusted for the same air pollutant during the other time windows based on multi-pollutant model.
* $p \leq 0.05$.

**Table S4.** Odds ratio (95%CI) of childhood autumn SAR to pollen for exposure to outdoor air pollution during different trimesters of pregnancy (n = 2598).

| | Multi-Pollutant Model † | | | | Multi-Pollutant + Window Model ‡ |
|---|---|---|---|---|---|
| | $PM_{10}+SO_2$ | $PM_{10}+NO_2$ | $SO_2+NO_2$ | $PM_{10}+SO_2+NO_2$ | |
| **1st trimester** | | | | | |
| $PM_{10}$ | 0.98 (0.66–1.47) | 0.97 (0.64–1.45) | — | 0.97 (0.64–1.45) | 0.96 ( 0.64–1.45) |
| $SO_2$ | 1.42 (1.02–1.98)* | — | 1.31 (0.81–2.11) | 1.31 (0.81–2.11) | 1.25 ( 0.77–2.02) |
| $NO_2$ | — | 1.58 (0.96–2.60) | 1.17 (0.58–2.39) | 1.19 (0.58–2.43) | 0.80 ( 0.32–2.01) |
| **2nd trimester** | | | | | |
| $PM_{10}$ | 0.93 (0.64–1.35) | 0.90 (0.62–1.31) | — | 0.91 (0.62–1.31) | 0.91 ( 0.63–1.33) |
| $SO_2$ | 1.37 (1.04–1.80)* | — | 1.22 (0.84–1.76) | 1.22 (0.84–1.77) | 1.18 ( 0.78–1.79) |
| $NO_2$ | — | 1.64 (1.04–2.60)* | 1.29 (0.71–2.36) | 1.33 (0.72–2.46) | 0.92 ( 0.41–2.06) |
| **3rd trimester** | | | | | |
| $PM_{10}$ | 0.81 (0.55–1.18) | 0.69 (0.48–1.01) | — | 0.67 (0.46–0.98)* | 0.64 ( 0.43–0.94)* |
| $SO_2$ | 1.22 (0.84–1.76) | — | 0.64 (0.37–1.14) | 0.60 (0.33–1.09) | 0.57 ( 0.30–1.07) |
| $NO_2$ | — | 2.19(1.37–3.49)*** | 2.71 (1.45–5.05)** | 3.35 (1.71–6.57)*** | 3.42 ( 1.57–7.45)** |

OR (95%CI) was estimated for an IQR increase in $PM_{10}$, $SO_2$, and $NO_2$.
Single-adjusted model: Models were adjusted for all covariates in Table 1.
† Multi-pollutant model: Models were further adjusted for the other air pollutants during the same time window based on single-adjusted model.
‡ Multi-pollutant + window model: Models were further adjusted for the same air pollutant during the other time windows based on multi-pollutant model.
* $p \leq 0.05$.
** $p \leq 0.01$.
*** $p \leq 0.001$.

**Table S5.** Pearson correlations between outdoor air pollutants during different time windows (n = 2598).

| Air Pollutants | $PM_{10}$ | $SO_2$ | $NO_2$ |
|---|---|---|---|
| **1 year before conception** | | | |
| $PM_{10}$ | 1.00 | 0.331** | −0.226** |
| $SO_2$ | | 1.00 | 0.676** |
| $NO_2$ | | | 1.00 |
| **Prenatal** | | | |
| **1st trimester** | | | |
| $PM_{10}$ | 1.00 | 0.409** | 0.154** |
| $SO_2$ | | 1.00 | 0.661** |
| $NO_2$ | | | 1.00 |
| **2nd trimester** | | | |
| $PM_{10}$ | 1.00 | 0.376** | 0.151** |
| $SO_2$ | | 1.00 | 0.594** |
| $NO_2$ | | | 1.00 |
| **3rd trimester** | | | |
| $PM_{10}$ | 1.00 | 0.522** | 0.286** |
| $SO_2$ | | 1.00 | 0.624** |
| $NO_2$ | | | 1.00 |
| **Entire pregnancy** | | | |
| $PM_{10}$ | 1.00 | 0.267** | −0.315** |
| $SO_2$ | | 1.00 | 0.482** |
| $NO_2$ | | | 1.00 |
| **Current** | | | |
| $PM_{10}$ | 1.00 | 0.898** | 0.907** |
| $SO_2$ | | 1.00 | 0.743** |
| $NO_2$ | | | 1.00 |

** Correlation is significant at the 0.01 level (2-tailed).
